# The methyltransferase domain of the Respiratory Syncytial Virus L protein catalyzes cap N7 and 2’-*O*-methylation

**DOI:** 10.1371/journal.ppat.1009562

**Published:** 2021-05-06

**Authors:** Priscila Sutto-Ortiz, Sergey Tcherniuk, Nina Ysebaert, Pravien Abeywickrema, Mathieu Noël, Alice Decombe, Françoise Debart, Jean-Jacques Vasseur, Bruno Canard, Dirk Roymans, Peter Rigaux, Jean-François Eléouët, Etienne Decroly

**Affiliations:** 1 Aix Marseille Université, CNRS, AFMB UMR 7257, Marseille, France; 2 Unité de Virologie et Immunologie Moléculaires, INRAE, Université Paris Saclay, Jouy en Josas, France; 3 Janssen Infectious Diseases and Vaccines, Beerse, Belgium; 4 IBMM, Université de Montpellier, ENSCM, CNRS, UMR 5247, Montpellier, France; Thomas Jefferson University, UNITED STATES

## Abstract

Respiratory syncytial virus (RSV) is a negative sense single-stranded RNA virus and one of the main causes of severe lower respiratory tract infections in infants and young children. RSV RNA replication/transcription and capping are ensured by the viral Large (L) protein. The L protein contains a polymerase domain associated with a polyribonucleotidyl transferase domain in its N-terminus, and a methyltransferase (MTase) domain followed by the C-terminal domain (CTD) enriched in basic amino acids at its C-terminus. The MTase-CTD of *Mononegavirales* forms a clamp to accommodate RNA that is subsequently methylated on the cap structure and depending on the virus, on internal positions. These enzymatic activities are essential for efficient viral mRNA translation into proteins, and to prevent the recognition of uncapped viral RNA by innate immunity sensors. In this work, we demonstrated that the MTase-CTD of RSV, as well as the full-length L protein in complex with phosphoprotein (P), catalyzes the N7- and 2’-*O*-methylation of the cap structure of a short RNA sequence that corresponds to the 5’ end of viral mRNA. Using different experimental systems, we showed that the RSV MTase-CTD methylates the cap structure with a preference for N7-methylation as first reaction. However, we did not observe cap-independent internal methylation, as recently evidenced for the Ebola virus MTase. We also found that at μM concentrations, sinefungin, a S-adenosylmethionine analogue, inhibits the MTase activity of the RSV L protein and of the MTase-CTD domain. Altogether, these results suggest that the RSV MTase domain specifically recognizes viral RNA decorated by a cap structure and catalyzes its methylation, which is required for translation and innate immune system subversion.

## Author summary

Respiratory syncytial virus (RSV) is responsible of infant bronchiolitis and severe lower respiratory tract infections in infants and young children, and the leading cause of hospitalization in children under one year of age. However, we still lack a vaccine and therapeutics against this important pathogen. The main enzymatic activities involved in RSV propagation are embedded in the Large (L) protein that contains the polymerase domain and also all the activities required for RNA cap structure synthesis and methylation. These post-transcriptional RNA modifications play a key role in virus replication because cap N7-methylation is required for viral RNA translation into proteins, and 2’-*O*-methylation hides viral RNA from innate immunity detection. Viral methyltransferase (MTase) activities are now considered potential antiviral targets because their inhibition might limit the virus production and strengthen early virus detection by innate immunity sensors. In this work, we compared the enzymatic activities of the MTase expressed as a single domain or in the context of the full-length L protein. We demonstrated that the MTase protein catalyzes the specific methylation of the cap structure at both N7- and 2’-*O*-positions, and we obtained the proof of concept that a S-adenosylmethionine analogue can inhibit the MTase activity of the L protein.

## Introduction

Respiratory syncytial virus (RSV) is a major seasonal human pathogen that infects nearly all children before two years of age and is the leading cause of severe lower respiratory tract diseases in newborn and young children [1]. It is estimated that RSV infection causes more than 60,000 deaths per year in children younger than 5 years old, mostly in developing countries [2] (http://perchresults.org). In addition, RSV is also recognized as a significant cause of severe respiratory infections in the elderly and high-risk adults [3]. Currently, the only clinical intervention is passive prophylaxis with the humanized monoclonal antibody palivizumab, used as a treatment to prevent hospitalization and severe RSV infections in high-risk populations [4]. Nevertheless, its use is restricted due to its high cost and modest efficacy [5]. Therefore, urgent efforts are needed to develop therapeutic agents against RSV infections, such as the small-molecule inhibitors currently in development to target the fusion process [6], or antiviral compounds against the replication/transcription machinery that is essential for viral propagation.

RSV is a filamentous enveloped, non-segmented negative-sense (NNS), single-stranded RNA virus that belongs to the *Orthopneumoviru*s genus, *Pneumoviridae* family, *Mononegavirales* order. This family also includes the human metapneumovirus (hMPV) that belongs to the *Metapneumovirus* genus and is another leading cause of acute respiratory infections in children. The *Mononegavirales* order comprises other important pathogens and sometimes deadly viruses, such as rabies and Ebola viruses [7].

The 15.2 kb RSV genome contains 10 genes that encode 11 proteins [6]. Three viral proteins are essential for RSV genome replication: the nucleoprotein (N), the large protein (L), and the phosphoprotein (P) [8,9]. The M2-1 protein is essential for viral transcription [10]. The N protein associates with viral RNA to protect it from cellular nucleases and recognition by the innate immune system [11,12]. The 250 kDa L protein harbors three conserved enzymatic domains: the RNA-dependent RNA polymerase (RdRp) domain, the polyribonucleotidyl-transferase (PRNTase or capping domain), and the methyltransferase (MTase) domain. The MTase domain is followed by the C-terminal domain (CTD), which is the most variable domain in NNS viruses. Despite the lack of obvious primary sequence conservation, it has been suggested that this domain, which is enriched in basic amino acids, forms a clamp with the MTase domain that allows the recognition of the RNA substrate for methylation [13]. Thus, the L protein of *Mononegavirales* participates in viral RNA synthesis, and also ensures its capping and methylation as well as the addition of the poly-A tail at the 3’ end of each transcript. The P protein, the main cofactor of the L polymerase, allows its interaction with the nucleoprotein-RNA complex [8,9,14,15]. mRNA capping and methylation are important co-transcriptional events during viral RNA biogenesis. The cap structure consists of a 7-methylguanosine (^m7^G) moiety linked to the first nucleotide of the transcript via a 5’-5’ triphosphate bridge [16]. The cap structure found at the 5’end of eukaryotic mRNAs has several important biological roles. For host cells mRNA, this modification i) ensures the protection of mRNA from degradation by 5’-3’ exoribonucleases; ii) allows mRNA translation by ensuring its efficient recognition by the eukaryotic translation initiation factor 4E (eIF4E) [17,18]; iii) directs pre-mRNA splicing and host mRNA export from the nucleus [19]; and iv) allows if missing, the recognition of foreign RNAs (including viral transcripts) as ‘non-self’ by recognition receptors, such as retinoic–acid inducible gene (RIG)-I [20–22]. In view of the pleiotropic role played by the RNA cap structure during infection, RNA capping enzymes and MTases are now considered as promising targets for the development of highly specific antiviral drugs [23,24]. Accordingly, mutation of MTase catalytic residues and molecules that block the cap MTase activity induce a strong antiviral response [25,26] and limit viral mRNA translation [24,27] in different viral families.

**Fig 1:**
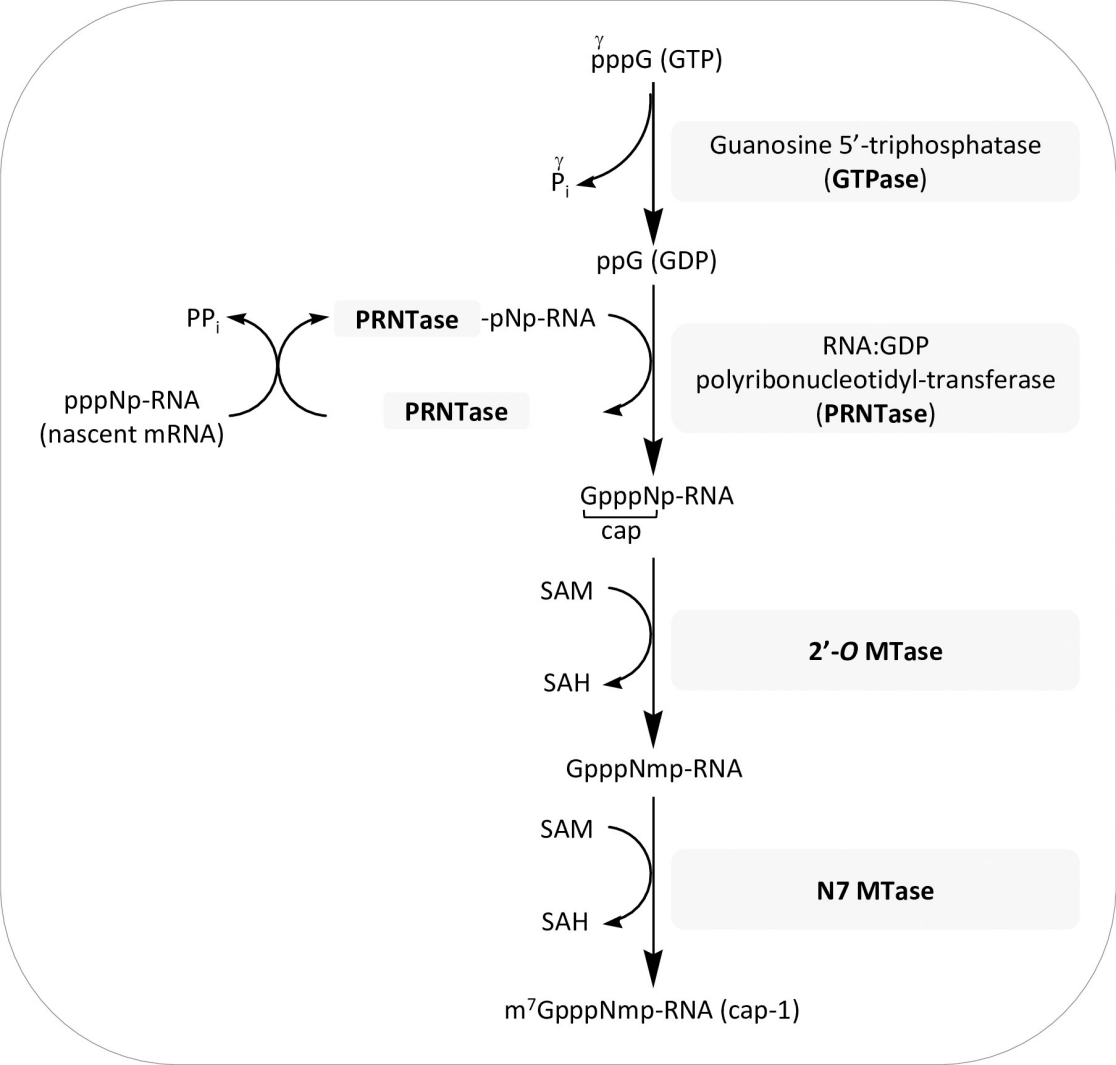
Schematic representation of the unconventional mechanism of mRNA cap formation by VSV L Protein. Possible mechanism described for the vesicular stomatitis virus (VSV) L protein where first hydrolyzes the γ-phosphate of guanosine-5’-triphosphate (GTP; Gppp) to yield guanosine diphosphate (GDP; Gpp) and inorganic phosphate (P_i_) [31]. Then, nascent mRNA transcripts that carry a triphosphate group at their 5’ end form a covalent adduct with the PRNTase histidine residue present in the conserved ‘HR’ motif of L, releasing pyrophosphate (PP_i_). The transfer of pNp-RNA to the GDP (in which N denotes the first transcribed nucleotide) forms the capped structure (GpppNp-RNA) that is subsequently methylated by S-adenosyl-L-methionine (AdoMet or SAM)-dependent methyltransferases (MTases) present in the C-terminal domain of L. First, the (nucleoside-2’-*O*)-methyltransferase (2’-*O*-MTase) transfers the methyl group from SAM to the first nucleotide of the nascent RNA, forming GpppNmp-RNA and releasing S-adenosyl homocysteine (SAH). Then, the cap structure is methylated on the guanine N7 position by the (guanine-N7-) methyltransferase (N7-MTase) to generate cap-1 RNA (m7GpppNmp-RNA).

The multifunctional L proteins of *Mononegavirales* have evolved independently from other known eukaryotic cap-synthesizing enzymes [28–30], and use an unconventional mechanism for mRNA cap synthesis that has been so far, only assessed for VSV [31,32] (Fig 1).

Conversely, MTase activities have been characterized in a larger set of mononegaviruses, including VSV [27], Ebolavirus [13,33] and hMPV [34]. The studies on the Ebola Sudan virus (SUDV) MTase and hMPV MTase revealed cap-dependent and cap-independent methyltransferase activities. For the SUDV MTase, a cap-independent 2’-*O*-MTase activity that targets internal adenosine residues was also described [33]. Recently, the 3.2-Å cryo-EM structure of RSV L bound to tetrameric P was described providing the first structural characterization of a viral phosphoprotein in complex with a viral L protein [35]. The structure reveals a striking tentacular arrangement of P, with each of the four monomers adopting a distinct conformation as it interacts with specific regions on L. In addition, the study provides the first structural information for the RdRp and capping domains of RSV L. However, the MTase and C-terminal domains of L protein are not visible in the cryo-EM map suggesting that the enzymatic activity of the RSV MTase can be regulated in the context of the full-length L-P protein complex. Although the structure of the RSV L protein in complex with P [35] as well as that of VSV [36,37] have brought important insights into the structure-function relation in the RSV RNA genome transcription and replication, the mechanism and the order of the reactions governing mRNA cap methylation are still elusive for viruses belonging to this family.

In this study, we expressed a 412-residue fragment of the RSV L protein that includes the MTase domain and the CTD (MTase-CTD) in bacteria. We found that the purified recombinant RSV MTase-CTD protein sequentially methylates the N7 and 2’-*O* positions of capped synthetic RNAs that mimic the RSV mRNA 5’end sequence. We observed similar enzymatic activities with purified full-length L protein co-expressed with P. However, we did not detect any internal methylation using capped and uncapped RNAs with both constructs. These findings suggest that the RSV MTase is a strict cap-dependent MTase.

## Results

### Cap-dependent MTase activity of RSV MTase-CTD

**Fig 2:**
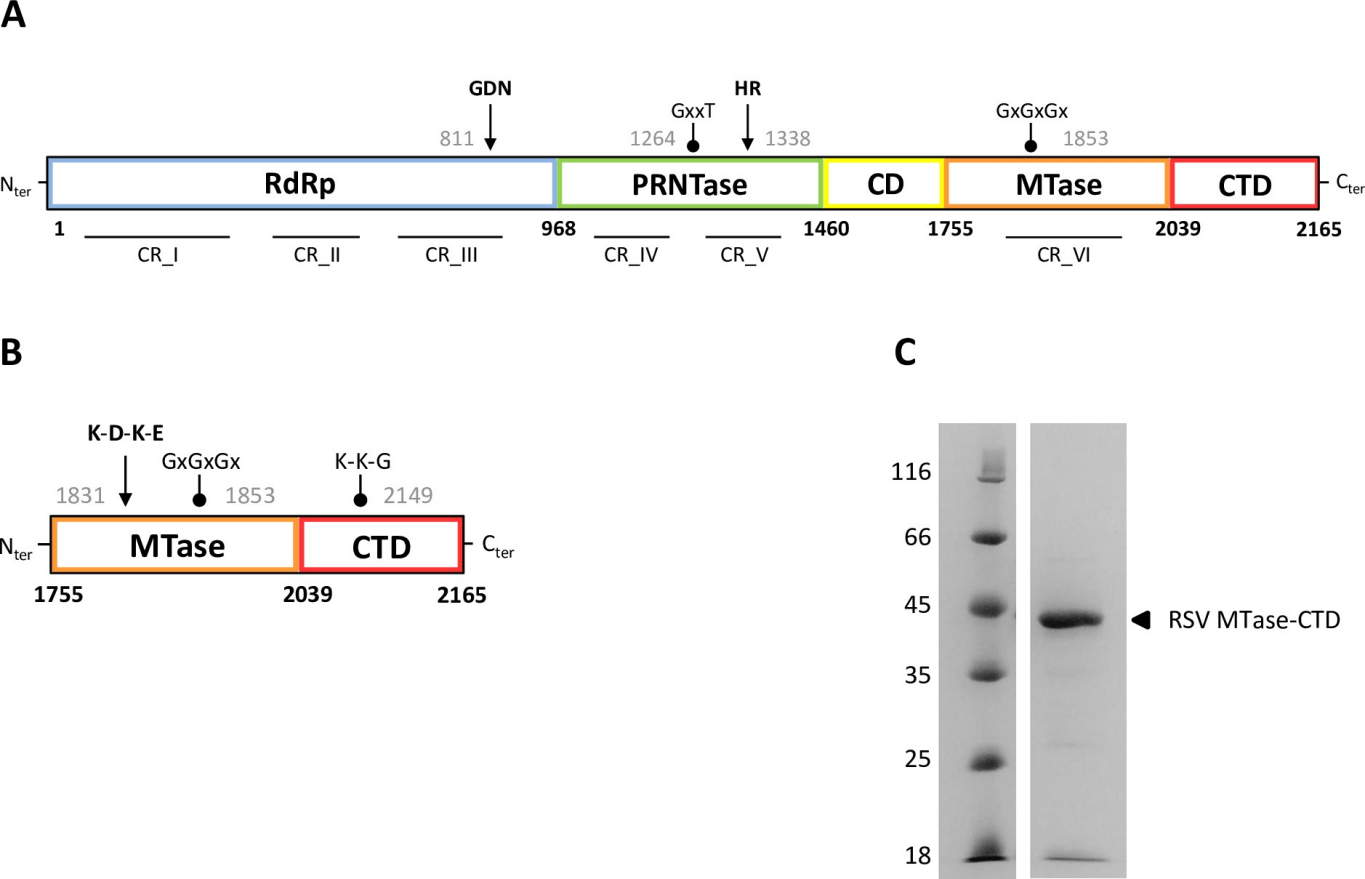
The recombinant RSV MTase-CTD protein. (**A**) Schematic representation of the domain organization of the RSV L protein. Blue, RNA-dependent RNA-polymerase (RdRp) domain; green, polyribonucleotidyl-transferase (PRNTase) domain; yellow, connector domain (CD); orange, methyltransferase (MTase) domain; red, C-terminal domain (CTD). Amino acid residue numbers indicate the functional domain boundaries. The conserved regions (CR) within the L proteins of non-segmented negative-strand RNA viruses are indicated. Arrows denote the position of active site residues required for function, and dots indicate conserved motifs with the starting amino acid residue number in gray. (**B**) The RSV MTase-CTD fragment was defined based on the alignment with the VSV L protein. Arrow indicates the K_1831_-D_1936_-K_1973_-E_2004_ catalytic tetrad, typical of 2’-*O*-MTases. Dots indicate conserved motifs with the starting amino acid residue number in gray: the GxGxGx SAM/SAH binding site motif in the MTase domain, and the K-K-G motif, reminiscent of eukaryotic GTases, in the CTD domain. (**C**) SDS-PAGE of the purified recombinant RSV MTase-CTD protein containing an N-terminal histidine tag (49.3 kDa). Molecular weights (in kilodaltons) of the ladder are shown on the left, and the MTase-CTD band is labeled on the right.

Based on the alignment of *Mononegavirales* MTases (Figs 2A and S1), we designed the RSV MTase-CTD construct that spans amino acids 1755 to 2165 and includes a 6 His-tag at the N-terminus. This construct corresponds to the MTase domain that includes the conserved K-D-K-E catalytic tetrad and the SAM/SAH binding GxGxGx motif, followed by the CTD required for substrate binding (Fig 2B). After expression in bacteria, we purified the recombinant RSV MTase-CTD protein to homogeneity by two steps of affinity chromatography followed by size-exclusion chromatography. SDS-PAGE analysis of the purified MTase-CTD protein (expected molecular weight of 49.3 kDa) showed a band of ≈45 kDa after Coomassie blue staining (Fig 2C). Matrix-Assisted Laser Desorption/Ionization Time-of-Flight (MALDI-TOF) analysis of the protein band confirmed its identity. We then assessed its MTase activity (optimum pH and the influence of Mg^2+^ ions) by incubating the purified MTase-CTD protein with a short-capped RNA substrate (GpppG-RSV_9_), in the presence of a radiolabeled methyl donor (^3^H-SAM). After separation of the radiolabeled capped RNAs from ^3^H-SAM by filter-binding assay (FBA), we determined the enzymatic activity by counting the radioactivity transferred to the RNA template. The MTase reaction showed a bell-shaped pH profile with an activity peak between pH 7.0 and 7.5 (S2A Fig). The RSV MTase-CTD activity was barely influenced by addition of 0.5 mM Mg^2+^ ions, and was only slightly decreased in the presence of 5 mM Mg^2+^ (S2B Fig).

**Fig 3:**
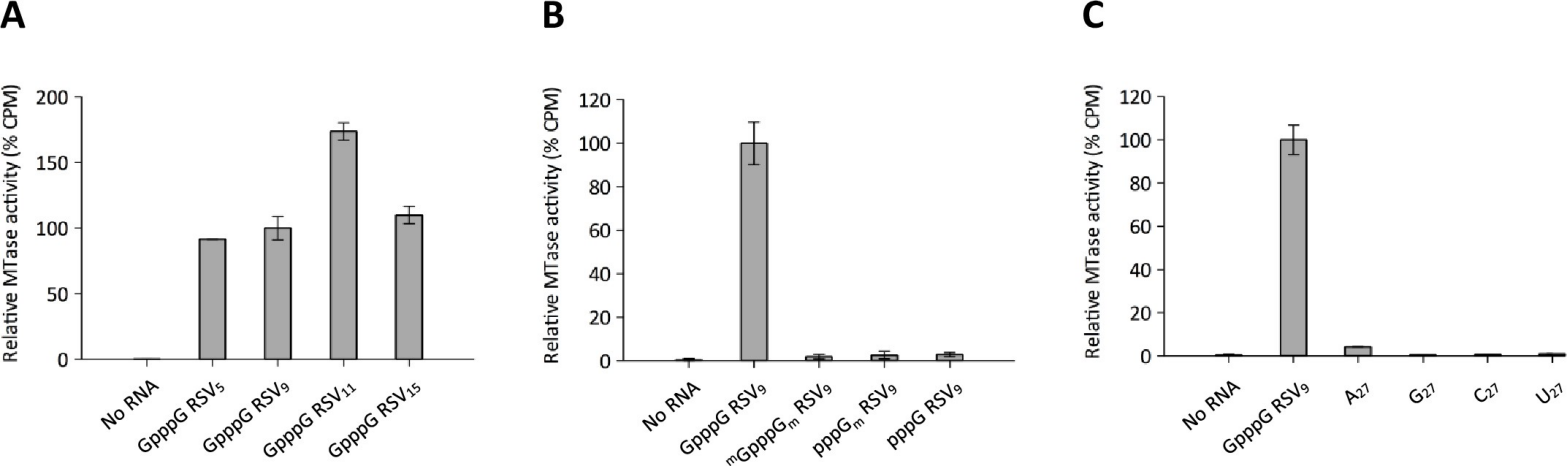
Cap-dependent MTase activity of the recombinant RSV MTase-CTD protein. (**A**) The transfer of tritiated methyl groups from S-adenosylmethionine (SAM) molecules to synthetic capped RNAs of different lengths (5-, 9-, 11- and 15- nucleotide-long), which mimic the 5’ end of RSV mRNA, was assessed by filter-binding assay. The RSV MTase-CTD was used at the concentration of 25 nM. Data are the mean values ± standard error of the mean (SEM) of three independent measurements. (**B**) MTase activity measurements using capped and uncapped RSV_9_ RNAs (GGG ACA AAA) methylated at specific positions. Values represent the mean ± SEM (*n* = 3). (**C**) MTase activity evaluation on synthetic, 27-nucleotide-long homopolymeric RNAs (HO-(G/C/U/A)_27_). Values were normalized to the activity on Gppp GGG-RSV_9_, and are the mean ± SEM (*n* = 3). CPM, count per minute.

We next determined the MTase-CTD substrate length preference by monitoring the transfer of the ^3^H-methyl group from SAM molecules to synthetic capped RNAs of increasing lengths (5-, 9-, 11- and 15- nucleotides) that correspond to the 5’ end of RSV mRNA. As the purified RSV MTase-CTD showed a dose-response MTase activity, for these experiments we used 25 nM of recombinant protein because the activity was detectable even at this low concentration. These experiments showed that the RSV MTase-CTD efficiently methylated the capped RNAs that mimic the 5’ end of RSV L mRNA [38], with preference for 11-nucleotide-long RNA (Fig 3A). We also evaluated its MTase activity on 9-nucleotide-long RSV_9_ RNA harboring a 5’ end cap-1 structure (^m^GpppG_m_) and on uncapped RNAs (pppG_m_, pppG). The RSV MTase-CTD exhibited cap-dependent methyltransferase activity, because GpppG-RSV_9_ was efficiently methylated, but not ^m^GpppG_m_-RSV_9_ (Fig 3B). Moreover, the RSV MTase-CTD protein did not methylate uncapped RNA substrates (pppG_m_-RSV_9_, pppG-RSV_9_), suggesting that the RSV MTase is a cap-dependent MTase. Finally, concerning the internal methylation activity, we observed that in our experimental conditions, the ^m^GpppG_m_-RSV_9_ RNA substrate and homopolymeric 27-mer RNAs (A_27_, G_27_, C_27_, U_27_) (Fig 3C) were barely methylated. Altogether, our results suggest that the RSV MTase catalyzes the cap structure methylation, and is scarcely involved in the internal RNA methylation activity.

**Fig 4:**
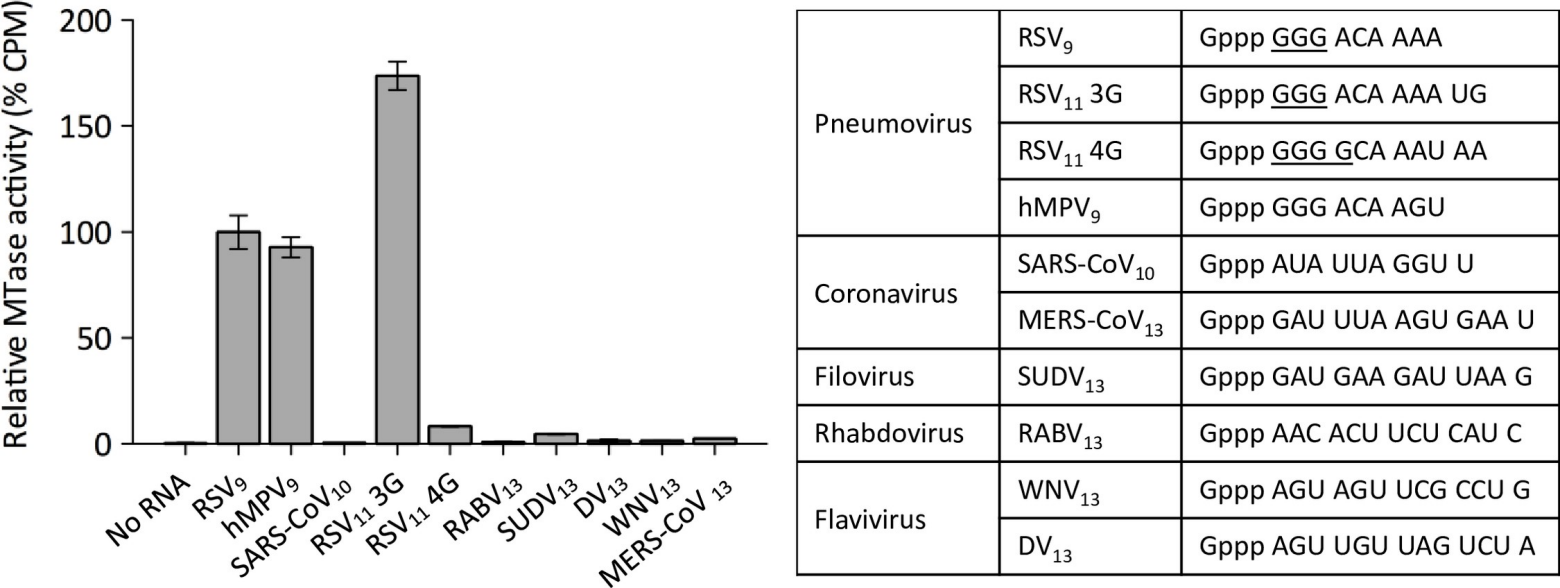
RSV MTase activity is sequence-specific. Substrate specificity was determined by filter-binding assay, using the synthetic capped RNA substrates with different 5’ end sequences and lengths (numbers after virus abbreviation) described in the table on the right. hMPV, human Metapneumovirus; SARS-CoV, Severe Acute Respiratory Syndrome Coronavirus; MERS, Middle East Respiratory Syndrome; SUDV, Sudan Ebolavirus; RABV, Rabies virus; WNV, West Nile virus; DV, Dengue virus. The RSV MTase-CTD was used at 25 nM. Values were normalized to the activity on Gppp GGG RSV_9_, and are the mean ± SEM (*n* = 3).

We then analyzed the substrate-sequence specificity using various synthetic capped RNAs with different viral 5’ end sequences (Fig 4). The RSV MTase-CTD displayed some sequence specificity, because it was mainly active on RSV_9_, RSV_11_ 3G and hMPV_9_ RNAs that share a GGG sequence at their 5’ end. Moreover, its MTase activity was 10 times lower in the presence of RSV sequences that contain four G nucleotides (RSV_11_ 4G) and mimic the 5’ end of the RSV P and N mRNAs [39,40]. Similarly, MTase-CTD was poorly active on RNA sequences that mimic the 5’ end of other viral genomes. Nevertheless, the MTase activity was slightly higher with substrates starting with Gppp G (SUDV_13_, MERS_13_) compared with Gppp AG (WNV_13_, DV_13_) and particularly Gppp AA (RABV_13_) or Gppp AU (SARS-CoV_10_).

To further characterize the RSV MTase activity, we mutated some conserved residues of the MTase catalytic site, in the SAM-binding site, and some basic residues supposed to participate in RNA binding (S1A Fig). After affinity chromatography purification, some variants were not expressed or were insoluble, particularly those harboring the K1831A, K1973A, K1999A, G1853S, G1857S, and L1878A substitutions. On the other hand, the MTase mutants that were successfully expressed and purified (S1B Fig) showed the expected molecular weight after SDS-PAGE analysis. The MTase activity of the RSV MTase-CTD protein was almost abolished by the D1936A and E2004A substitutions within the K-D-K-E tetrad (S1C Fig). This demonstrated that the catalytic tetrad is essential for the MTase activity. Serine substitution of G residue (G1855S) in the GxGxGx SAM/SAH binding-site motif and alanine substitution of residues R1820A, E1938A and S1998A involved in RNA-binding also strongly inhibited the MTase activity of the RSV MTase-CTD.

### The RSV MTase-CTD catalyzes N7- and 2’-*O*-methylation of the RNA cap structure

**Fig 5:**
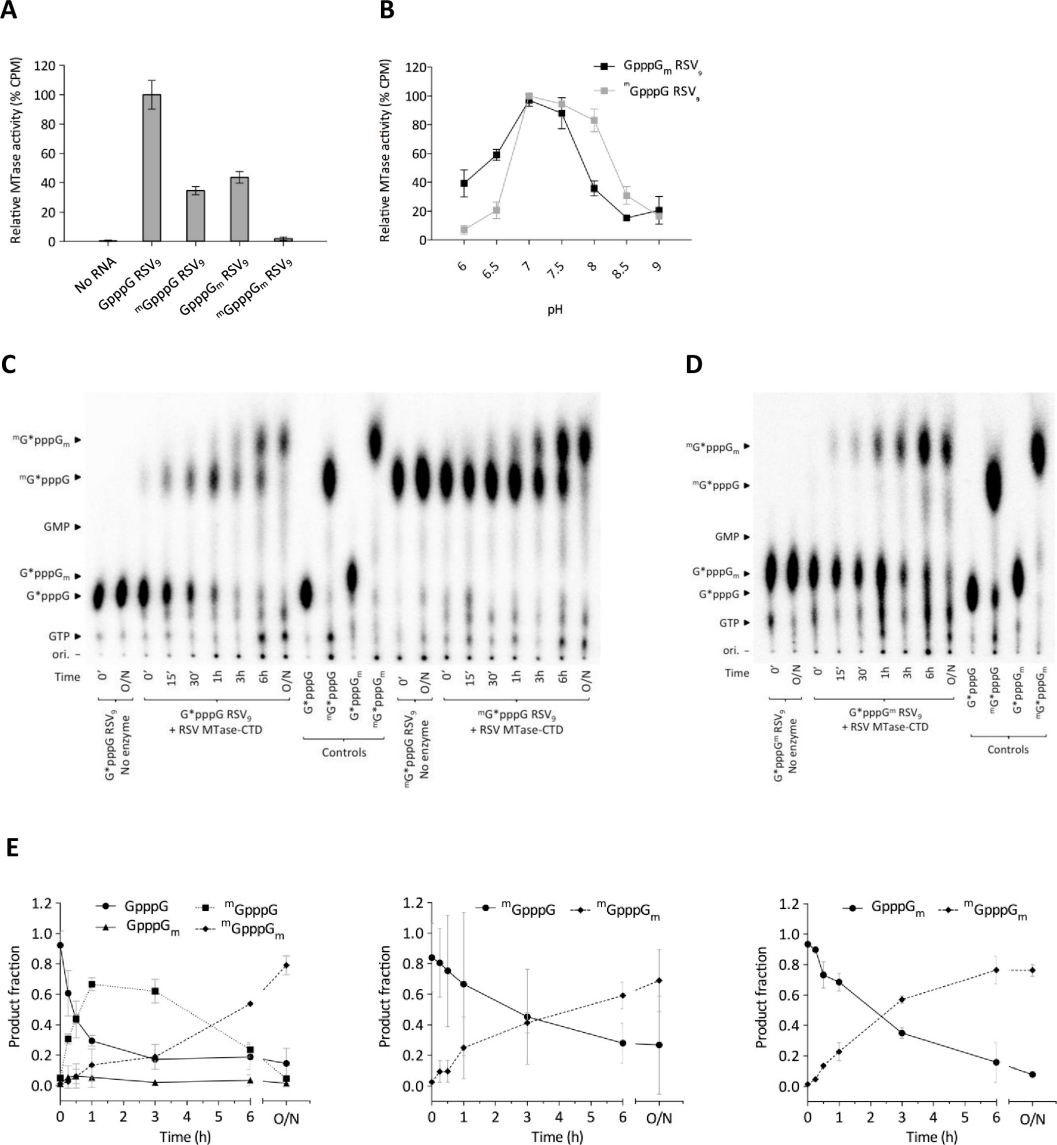
N7- and 2’-*O*-methylation activity of the RSV MTase-CTD protein. (**A**) MTase activity measurements by filter-binding assay using capped RSV_9_ RNAs (GGG ACA AAA). Values were normalized to the activity on Gppp GGG-RSV_9_ and are the mean ± SEM (*n* = 3). (**B**) Time-course of N7- and 2’-*O*-methylation activities on GpppG_m_ and ^m^GpppG RSV_9_ RNAs, respectively, by the RSV MTase-CTD protein (25 nM) measured by filter-binding assay at different pH (from 6.0 to 9.0). The plotted values were obtained after 3 h incubation at 30°C. Values were normalized to the maximum activity on each substrate and are the mean ± SEM (*n* = 3). (**C, D**) Thin-layer chromatography analysis of cap structures of control RNAs and substrates (G*pppG (C left), ^m^G*pppG (C right), G*pppG_m_ (D)) incubated with the RSV MTase-CTD (* indicates G(^32^P)-labeled). Nucleotides 2–9 were removed by nuclease P1 digestion and caps were separated using 0.65 M LiCl as mobile phase. Controls (G*pppG, G*pppG_m_, ^m^G*pppG, ^m^G*pppG_m_) were obtained using the pppG-RSV_9_ substrate and vaccinia virus MTases that specifically methylate caps at the N7 or 2’-*O* positions. The RSV MTase-CTD was used at the concentration of 25 nM. O/N:overnight. (**E**) Densitometry quantitations of TLC analysis expressed as a ratio of the substrate consumption (G*pppG (left), ^m^G*pppG (middle), G*pppG_m_ (right)) and product formation over time. Values are the mean ± SEM (n = 3).

To further characterize the methylation catalyzed by the RSV MTase-CTD, we compared the MTase activity on synthetic 9-mer RSV_9_ RNAs with different cap structures (GpppG, ^m^GpppG, GpppG_m_ and ^m^GpppG_m_). Compared with the GpppG-RSV_9_ RNA (Fig 5A), the RSV MTase-CTD methylation activity was reduced by ~50% in the presence of the RNAs with the monomethylated ^m^GpppG-RSV_9_ and GpppG_m_-RSV_9_ cap structures, and was not detectable when both the N7 and 2’-*O* positions of the cap were methylated (^m^GpppG_m_-RSV_9_). This suggested that the RSV MTase-CTD protein catalyzes N7 and 2’-*O*-methylation of the cap structure. We then determined the optimum pH of each MTase activity by measuring the transfer of the ^3^H-methyl group from SAM molecules to the GpppG_m_-RSV_9_ and ^m^GpppG-RSV_9_ substrates. The curves adopt a bell-shaped profile with an optimum at pH 7 to 7.5 for the N7-MTase activity and a broader pH range from pH 7 to 8 for the 2’-*O*-MTase activity (Fig 5B).

We next confirmed by thin-layer chromatography (TLC) the nature of the methylations catalyzed on the RSV_9_ RNA cap structure. To this aim, pppG-RSV_9_ RNA was incubated with the vaccinia virus GTase/N7-MTase capping system in the presence of [α-^32^P]-GTP and SAM in the reaction buffer, to produce GpppG-RSV_9_ or ^m^GpppG-RSV_9_. A time-course experiment was then performed using the GpppG-RSV_9_ RNA as substrate and the RSV MTase-CTD protein. We digested the reaction products with P1 nuclease before TLC analysis of the cap structure. After 15 min, GpppG-RSV_9_ was converted into ^m^GpppG-RSV_9_ (30.7±4.7%) or GpppG_m_-RSV_9_, (1.5±0.5%) (Fig 5C left). After a longer incubation period, we could detect the double methylated product (^m^GpppG_m_), and the products corresponding to the monomethylated cap structures (^m^GpppG and GpppG_m_) disappeared progressively, as expected. After 6 hours of incubation, GpppG-RSV_9_ was almost entirely converted into ^m^GpppG_m_-RSV_9_. Similarly, when we used the ^m^GpppG-RSV_9_ RNA substrate (Fig 5C right) or GpppG_m_-RSV_9_ RNA (Fig 5D), we detected the ^m^GpppG_m_ product after 15 min of reaction, demonstrating that in our experimental conditions, the 2’-*O-*methylation can occur after N7 methylation of the guanosine residue of the cap structure.

In order to estimate kinetic parameters of the cap methylation process, time-course assays were performed in triplicates, and we quantified by densitometry the substrate and the methylated species detected on TLC. The quantification shows that when the MTase-CTD protein is incubated with GpppG-RSV_9_ substrate (Fig 5E left), the estimated initial velocities (V_i_) for the generation of ^m^GpppG-RSV_9_ or GpppG_m_-RSV_9_ product are V_i_ = 0.948±0.119 and V_i_ = 0.078±0.026 product formation/h, respectively. The N7 methylation is about 12 fold faster than the 2’-*O* methylation in our experimental conditions. The subsequent 2’-*O* methylation of ^m^GpppG-RSV_9_ then takes place resulting in the ^m^GpppG_m_-RSV_9_ product for which we obtained a V_i_ = 0.132±0.007 product formation/h. In addition, the V_i_ estimated for the generation of ^m^GpppG_m_-RSV_9_ product from either ^m^GpppG-RSV_9_ (Fig 5E middle) or GpppG_m_-RSV_9_ (Fig 5E right) substrate provides a similar value of V_i_ = 0.256±0.025 and V_i_ = 0.234±0.013 product formation/h, respectively. Altogether our results suggest that the cap methylation by the RSV MTase-CTD occurred at the N7 and also 2’-*O* positions, with a preference for N7 methylation as first reaction step. In addition it is likely that the 2’-*O* methylation is the limiting step for the capping reaction carried out by the MTase-CTD protein.

### The MTase domain and the RSV L-P complex display similar N7 and 2’-*O*-MTase activities

**Fig 6:**
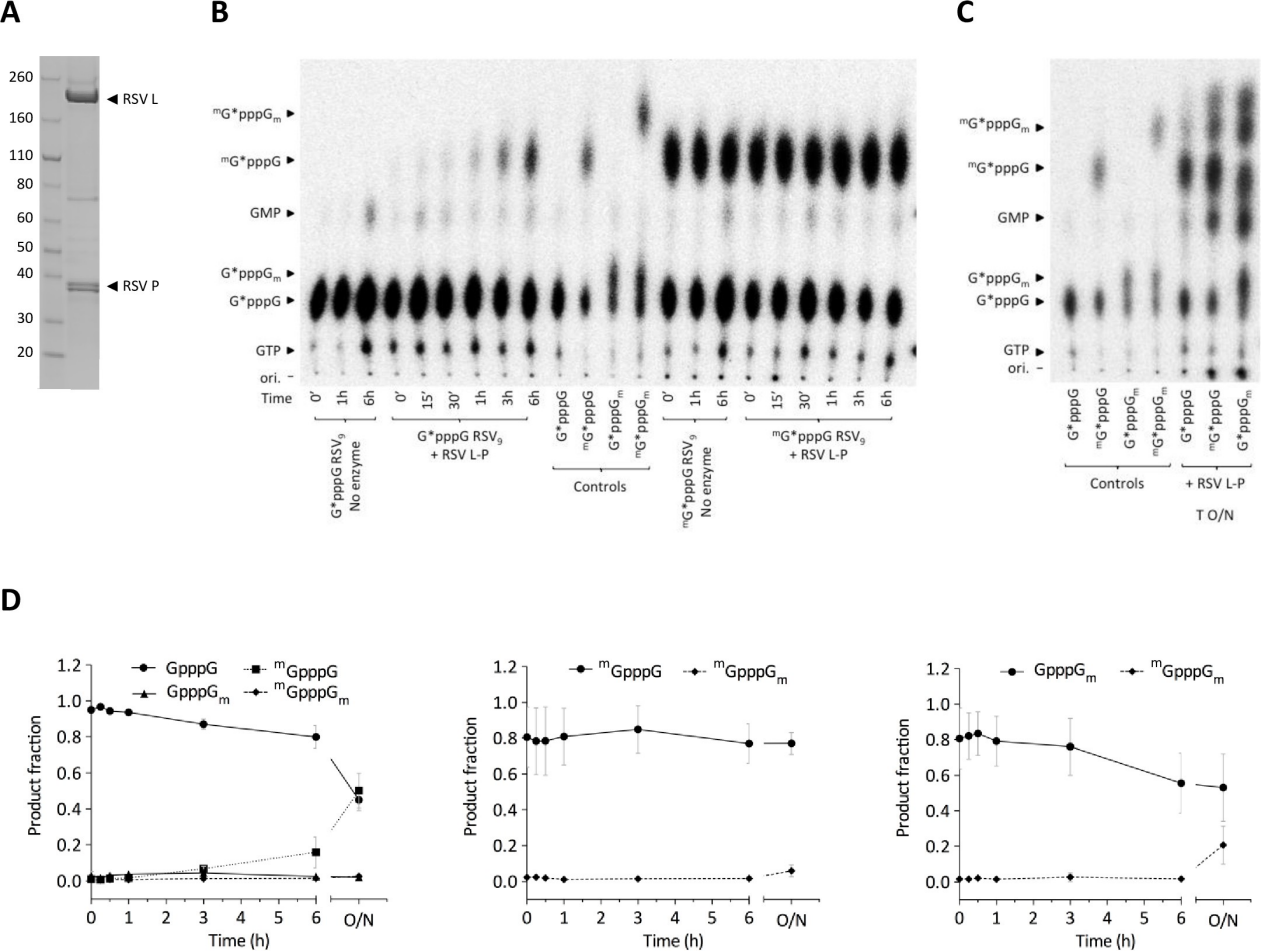
MTase activity of the RSV L-P complex. (**A**) SDS-PAGE of the purified RSV L-P complex. Molecular weights (in kilodaltons) of the ladder are shown on the left, and the L and P bands are labeled on the right. (**B**) Time-course analysis of methylation by thin-layer chromatography of the cap structures of control RNAs and substrates (G*pppG and ^m^G*pppG) incubated with RSV L-P (* indicates G(^32^P)-labeled) for up to 6 h at 30°C. (**C**) After overnight (O/N) incubation, nucleotides 2–9 were removed by nuclease P1 digestion and caps were separated using 0.65 M LiCl as mobile phase. Controls (G*pppG, G*pppG_m_, ^m^G*pppG, ^m^G*pppG_m_) were obtained using the pppG-RSV_9_ substrate and vaccinia virus MTases that specifically methylate caps at the N7 or 2’-*O* positions. The RSV L-P complex was used at the concentration of 100 nM. (**D**) Densitometry quantitations of TLC analysis expressed as a ratio of the substrate consumption (G*pppG (left), ^m^G*pppG (middle), G*pppG_m_ (right)) and product formation over time. Values are the mean ± SEM (n = 3).

Cryo-EM studies indicate that the MTase domain of L protein can accommodate different positions, suggesting that its enzymatic activity can be regulated in the context of the full-length L protein or in the L-P protein complex [35]. To further characterize the MTase activities of the purified L-P complex (Fig 6A), we analyzed the nature of the cap structure methylation by TLC analysis. In this time-course experiment, we incubated RNAs carrying different types of ^32^P-radiolabeled cap structures with the RSV L-P complex, and then digested the reaction products with P1 nuclease. TLC analysis of the released cap structures methylated at position N7 (cap-0), 2’-*O*, or both (cap-1) showed that the RSV L-P complex methylated GpppG-RSV_9_ at the N7 position, as indicated by the presence of ^m^GpppG-RSV_9_ after 30 min of reaction (Fig 6B left). However, after 6 hours incubation, we could barely detect the double methylated ^m^GpppG_m_-RSV_9_ product. We obtained similar results when we incubated the RSV L-P complex with the ^m^GpppG-RSV_9_ (Fig 6B right) or GpppG_m_-RSV_9_ substrates, suggesting that 2’-*O-*methylation is a limiting step in our experimental conditions. After overnight incubation of the reaction mixtures, we could observe a faint band corresponding to the ^m^GpppG_m_ cap structure, indicating that 2’-*O*-methylation occurs (Fig 6C), but that its kinetics were slower when using the L-P complex than the RSV MTase-CTD alone. These results indicate that the RSV MTase in the context of the L-P complex catalyzes more efficiently N7 methylation of capped RNA than 2’-*O-*methylation. These observations were confirmed by the densitometry quantitation of the TLC analysis (Fig 6D), but the kinetic parameters were not determined due to the low amount of reaction product detected in our experimental conditions.

### RSV MTase inhibition by SAM and Cap analogues

As the RSV MTase uses SAM as methyl donor to catalyze cap N7 and 2’-*O*-methylation, we wondered whether SAM or cap analogues could competitively inhibit the RSV MTase enzymatic activity. We first determined whether cap analogues inhibit the RSV MTase activity. We incubated N7-methylated (^m^GpppA, ^m^GDP) and unmethylated (GpppA, GpppG) cap analogues with the RSV MTase-CTD, and determined the methylation of GpppG-RSV_9_-RNA by FBA as previously described. The cap analogue GpppG at 50 μM inhibited by 50% the activity of the RSV MTase-CTD (S3A Fig), whereas the GpppA (S3A Fig) and the N7-methylated cap analogues (S3B Fig) showed lower inhibitory activity.

**Fig 7:**
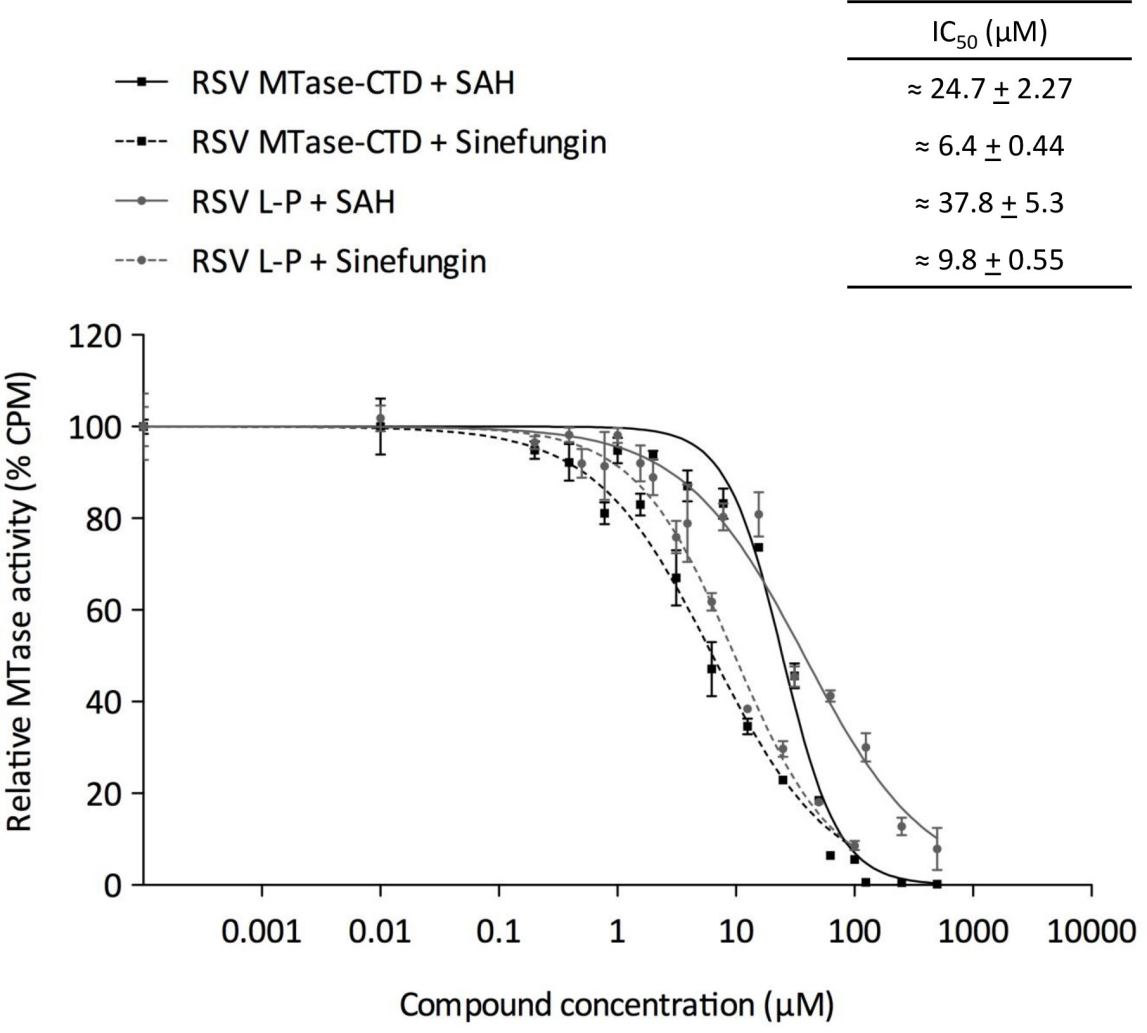
Inhibition of the MTase activity by SAM analogues. Increasing concentrations of SAH or sinefungin (previously dissolved in water) were incubated with 25 nM RSV MTase-CTD in a reaction mixture (40 mM Tris-HCl, pH 7.5, 2 μM SAM and 0.1 μM ^3^H-SAM) in the presence of 0.7 μM of GpppG-RSV_9_ synthetic RNA, and with 60 nM RSV L-P complex in a reaction mixture (40 mM Tris-HCl, pH 7.5, 0.17 μM SAM and 0.8 μM ^3^H-SAM) in the presence of 1.8 μM synthetic RNA (GpppG-RSV_9_). Reactions were incubated at 30°C for 3 h. Values were normalized and fitted with Prism (GraphPad software) using the following equation: Y = 100/(1+((X/IC_50_)^Hillslope)) (n = 3; mean value ± SEM).

We next measured by FBA the inhibition of the MTase activity by SAM/SAH by performing a dose-response assay using increasing concentrations of sinefungin and SAH (the methylation reaction by-product). After activity normalization (typical dose-response inhibition curve in Fig 7), deduction of the half-maximal inhibitory concentration (IC_50_) by Hill slope curve-fitting [equation: Y = 100/(1+((X/IC_50_)^Hillslope))] showed IC_50_ values of 6.4 μM and 24.7 μM for sinefungin and SAH, respectively. To confirm the robustness of the enzymatic assay performed with the RSV MTase-CTD protein, we also carried out a dose-response inhibition assay using the RSV L-P complex in the presence of sinefungin and SAH. The IC_50_ values were in the same range (9.8 μM and 37.8 μM for sinefungin and SAH, respectively), confirming that the MTase domain can be inhibited by SAM analogues, which opens promising perspectives for the development of high-throughput screening assays to identify new antiviral compounds. However, antiviral activity with sinefungin performed in cellular assay did not show inhibition activity on replication of RSV in HeLa cells before toxicity. Of note, the antiviral activity of sinefungin has been shown to inhibit VSV replication in BHK cells using plaque assay, with a half maximal effective concentration (EC_50_) of 220 μM [41].

## Discussion

Mononegaviruses share a similar genetic organization in which the L protein orchestrates the replication/transcription and capping activities. However, the MTase activity specificity might vary according to the virus family. Indeed, it was shown that the Ebola virus MTase catalyzes internal methylation of RNA in addition to cap methylation, whereas the hMPV MTase mainly catalyzes N7 and 2’-*O*-methylation of the cap structure present at the 5’end of viral RNA [33,34]. So far, descriptions in the literature involving the activity of RSV L have been assessed as independent structural fragments of the polymerase with dimerization tags resulting in trans-complementation of RdRp bioactivity [42]. Nevertheless, to date, information on RSV RNA methylation mechanisms is missing. In this work, we produced and purified the soluble RSV MTase-CTD protein, and we analyzed *in vitro* its enzymatic activity using different RNA substrates. We found that the RSV MTase-CTD is active at 25 nM, unlike the MTase domain purified from other NNS viruses, such as Ebola virus [33] or hMPV [34], that are active at μM concentrations. The difference in the amount of protein required for MTase activity determination could be explained by the fact that Ebola virus [33] and hMPV MTases [34] were purified using astringent conditions (*i*.*e*. arginine) to ensure their solubility, whereas the RSV MTase-CTD was purified under mild conditions that are probably more favorable for MTase activity assay. Mutagenesis studies demonstrated that the RSV MTase activity is specific and is strongly impaired by single alanine substitution of residues belonging to the catalytic site (K-D-K-E), as well as mutation of conserved residues that are thought to participate in SAM or RNA-binding. Similar mutation effects were previously described for the Ebola SUDV [33] and hMPV [34] MTases.

We observed that the RSV MTase displays sequence preference because the protein methylates more efficiently capped RNA starting with GGG, a sequence that is present at the 5’ end of most RSV mRNAs [39]. Conversely, we did not detect any obvious effect of RNA length (fragments between 5 and 15 nucleotides in length were tested). Moreover, the presence of a fourth G at the 5’ end of RNA reduced the MTase activity. This was unexpected because some RSV mRNAs (from P and N proteins) have been inferred to start with a GGGG sequence [40], but their exact 5’ end sequence is not formally demonstrated. Therefore, the methylation of such RNAs might occur co-transcriptionally, on the nascent viral RNA synthetized by the RdRp domain of the L protein, to ensure their efficient methylation. The sequence specificity of the RSV MTase domain is also reflected by its low methylation activity in the presence of RNAs starting with sequences that mimic the 5’ end of other viruses (coronavirus, filovirus, rhabdovirus and flavivirus). These findings suggest that the RSV MTase has a specific recognition mechanism based on the nucleotide sequence present at the 5’ end of capped RSV RNA.

We also investigated whether the RSV MTase catalyzes only the specific methylation of cap structures or also additional internal RNA methylation, as previously reported for Ebola and flaviviruses (Dengue, Zika) [13,33,43,44]. We could barely detect the RSV MTase-CTD enzymatic activity in the presence of homo- and hetero-polymeric uncapped RNA substrates, as well as of ^m^GpppG_m_-RSV_9_-RNA, suggesting that no internal methylation occurs in our experimental conditions. This is strikingly different from what reported for the Ebola SUDV MTase that displays mainly internal methylation activity *in vitro* [33], and for the hMPV and Ebola virus MTases that catalyze the 2’-*O*-methylation of the first ribose nucleotide in the absence of any cap structure [34]. Thus, the cap or RNA recognition mechanism might be specific for the different NNS MTases that share a conserved MTase domain, but that have a CTD (involved in RNA recognition) of different length and sequence. Although the hMPV MTase CTD structure revealed that this MTase lacks any obvious cap-binding site [34], such as the one present in flaviviruses, coronaviruses and vaccinia virus [24,45,46], the RSV CTD might have evolved to accommodate preferentially cap structures. Additional studies are needed to determine the molecular bases of cap recognition by the RSV L protein.

Concerning the cap-dependent MTase activity, we characterized how the cap structure is methylated in our *in vitro* assay. Methyl transfer to RNA substrates that mimic the conserved start sequence of RSV 5’ transcripts occurred at the 2’-*O* position and also N7 position in the FBA assay, but N7 methylation was catalyzed more efficiently in the time-course assay performed with the MTase-CTD construct. We obtained similar results (*i*.*e*. higher N7 MTase activity) also when we tested the RSV MTase in the context of the L-P complex. Nevertheless, we observed that the kinetics of methylation by the MTase in the L-P complex are slower compared to the MTase-CTD protein. This suggests that the accessibility of the MTase domain active site is decreased in the L-P complex. In these experimental conditions, N7-methylation occurred first, and 2’-*O* activity was only visible after overnight incubation. The time-course analysis of cap structure methylation by RSV MTase-CTD confirmed that there is a preferred sequence of methylation events. Altogether, these results suggest that N7-methylation occurs before the first RNA nucleotide (N1) 2’-*O*-methylation. This order of methylation events is different from what is described for the cap methylation mechanism of most mononegaviruses (VSV, hMPV, Ebola virus) in which 2’-*O*-methylation was proposed to precede N7 methylation [31–34,47]. Conversely, this mechanism is reminiscent of what is observed in eukaryotes (metazoans) and other viral systems, such as vaccinia virus [48–51], where the guanosine from the cap structure is methylated at the N7 position before methylation at the 2’-*O* position of N1 [52–55]. Regardless of the methylation sequence, the currently available mononegavirus structures suggests that the MTase domain possesses an active center (K-D-K-E) and a single SAM binding pocket involved in the methyl transfer reaction to two distinct acceptor positions with different chemical characteristics and conformational contexts. To achieve this, the mRNA cap substrate must accommodate two different positions: one with the guanine-N7 position and the other with the nucleoside-2’-*O* position facing the SAM methyl group [56,57].

In conclusion, this work shows that RSV harbors a SAM-dependent MTase activity to catalyze N7- and 2’-*O*-methylations of viral mRNA. Inhibition of such activities could limit viral replication and stimulate the antiviral response. We demonstrated that SAM analogues, such as sinefungin, inhibit the RSV MTase enzymatic activity of the L-P complex and of the MTase-CTD domain with similar IC_50_ values. The biochemical characterization and enzymatic assays developed in this work pave the way for the development of new antiviral strategies to target the RSV MTase in order to limit the viral replication or potentiate the host antiviral response.

## Materials and methods

### Cloning and expression

The codon-optimized RSV MTase-CTD synthetic gene (Genscript) was cloned in a modified pGEX-4T-3 vector with an N-terminal 6x His-tag for bacterial expression. Mutants were generated by PCR amplification using the *Pfu* DNA polymerase kit (Promega) and primers carrying the mutations (S2 Table). Proteins were produced in *Escherichia coli* chaperone competent pGro7/BL21 cells (Takara). Cells were grown in Luria broth with L-arabinose (1 g/L) at 30°C until the OD_600_ value reached 0.6. Protein expression was induced with 20 μM IPTG at 16°C, overnight. Bacteria were then spun down (8000 rpm, 4°C, 10 min) using a Sorval Lynx 6000 centrifuge (Thermo Scientific), and pellets were stored at -80°C.

### Protein purification

The MTase-CTD construct was purified as follows. Cell pellets from 1 L bacterial culture were resuspended in 30 ml lysis buffer (50 mM Tris-HCl pH 7.5, 500 mM NaCl, 20 mM imidazole, 10% glycerol, 2 mM PMSF, 2 mM β-mercaptoethanol, 1x BugBuster (Merck; 10x solution), 1% Triton X-100, 10 μg/mL DNase I, 0.25 mg/mL lysozyme, 1 tablet antiprotease cocktail (Bimake)), incubated at 4°C for 30 min, and sonicated. After cell lysate clarification (20,000 rpm, 4°C, 30 min), the supernatant was incubated with CoNTA resin (Thermo Scientific; 0.5 mL/L culture) at 4°C with gentle shaking for 1 h. Beads were transferred to a 25 mL column and washed with 2x10 mL of the following washing (W) buffers: W1 (50 mM Tris-HCl pH 7.5, 1 M NaCl, 20 mM imidazole, 10% glycerol, 1% Triton X-100, 2 mM β-mercaptoethanol), W2 (50 mM Tris-HCl pH 7.5, 50 mM NaCl, 20 mM imidazole, 10% glycerol, 1% Triton X-100, 2 mM β-mercaptoethanol), and W3 (50 mM Tris-HCl pH 7.5, 500 mM NaCl, 20 mM imidazole, 10% glycerol, 2 mM β-mercaptoethanol). Proteins were eluted with W3 buffer containing 250 mM imidazole and dialyzed overnight in heparin buffer A (20 mM Tris-HCl pH 7.5, 200 mM NaCl, 10% glycerol, 1 mM DTT). A HiTrap heparin column (GE Healthcare) was used as second purification step, and proteins were eluted with a linear gradient of heparin buffer B (20 mM Tris-HCl pH 7.5, 1.5 M NaCl, 10% glycerol, 1 mM DTT). Size-exclusion chromatography (SEC) was used as third purification step with a Superdex 200 10/300 GL column (GE Healthcare) in SEC buffer (50 mM Tris-HCl pH 7.5, 500 mM NaCl, 10% glycerol, 1 mM DTT). The RSV MTase-CTD was concentrated using Vivaspin ultrafiltration units (Sartorius), and stored at -20°C in SEC buffer containing 40% glycerol. Contrary to the hMPV and Ebola MTases that were purified in presence of arginine to increase their solubility, the RSV MTase was soluble in absence of arginine.

The L-P construct was produced and purified as previously described [35]. Briefly, the human RSV L protein (strain A2) with an N-terminal dual StrepTag and the RSV P protein (strain A2) with a C-terminal 6x His-tag were co-expressed in Sf9 insect cells using the pFASTbac Dual transfer vector. The L-P complex was purified with two affinity chromatography steps, followed by size-exclusion chromatography [35].

### MTase activity assay

The MTase activity was measured using a filter-binding assay, performed by combining 25 nM of RSV MTase-CTD with 0.7 μM of synthetic RNAs, 2 μM SAM, and 0.33 μM ^3^H-SAM (Perkin Elmer) in 40 mM Tris-HCl pH 7.5. After 3-h incubation at 30°C, reactions were quenched by 10-fold dilution in ice-cold 100 μM SAH, and samples were transferred to DEAE filtermats (Perkin Elmer) using a Filtermat Harvester (Packard Instruments). The RNA-retaining mats were washed twice with 10 mM ammonium formate (pH 8.0), twice with water, and once with ethanol. They were then soaked with liquid scintillation fluid for measuring ^3^H-methyl transfer to the RNA substrates using a Wallac MicroBeta TriLux Liquid Scintillation Counter (Perkin Elmer).

### Synthesis of RNA substrates

RNA sequences were chemically synthesized on a solid support using an ABI 394 automated synthesizer with 2’-*O*-pivaloyloxymethyl 3’-*O*-phosphoramidite ribonucleosides and 2’-*O*-methyl 3’-*O*-phosphoramidite guanosine (ChemGenes Corp., USA) [58]. After RNA assembly, the 5’-hydroxyl group was phosphorylated and the resulting *H*-phosphonate derivative was oxidized and activated into a phosphoroimidazolidate derivative to react with pyrophosphate [59] or GDP, giving pppRNA or GpppRNA, respectively. After deprotection and release from the solid support, RNA substrates were purified by IEX-HPLC (>95% pure) and their identity was confirmed by MALDI-TOF spectrometry. N7-methylation of the cap structure was performed using human (guanine-N7)-MTase [60].

### Thin-layer chromatography (TLC) analysis of cap structures

Radioactive capped G*ppp RNAs (in which the asterisk indicates the [^32^P]-labelled phosphate) were synthesized by incubating ppp RNA (10 μM) with the vaccinia virus capping enzyme (New England Biolabs) in the presence of 1.65 μCi [α-^32^P]-GTP (Perkin Elmer). Capped RNAs were purified with MicroSpin G-25 columns (GE Healthcare) and StrataClean resin (Agilent) before incubation with the RSV MTase-CTD. After methylation, capped RNAs were purified by precipitation in 3 M sodium acetate supplemented with 1 μg μL^-1^ of glycogen (Thermo Scientific), followed by digestion with 1 U of nuclease P1 (Sigma) at 37°C for 1 h. Digested products were spotted onto polyethylenimine cellulose TLC plates (Macherey Nagel), and resolved using 0.65 M LiCl as mobile phase. After autoradiography, the radiolabeled caps released by the nuclease P1 were visualized using an Amersham Typhoon Biomolecular Imager (GE Healthcare). The quantitative analysis was performed using the ImageQuant TL software (GE Healthcare). The initial velocity was determined by linear regression analysis of the linear phase of a product build-up using GraphPad Prism version 5.0.

## Supporting information

S1 Fig Mutagenesis of the RSV MTase-CTD protein and effect on N7- and 2’-*O*-methylation. (**A**) Amino acid sequence alignment of the C-terminal portion of the indicated L proteins showing the amino acid residues of the catalytic tetrad (K-D-K-E) and the amino acid residues thought to participate in SAM and RNA-binding that were mutated. The amino acid sequences of the L protein from RSV strain A2 (P28887), human metapneumovirus (Q91L20), Zaire ebolavirus strain Mayinga-76 (Q05318) and vesicular stomatitis virus Indiana strain (P03523.2) were aligned using Seaview (Gouy *et al*., 2010) and ESPript (http://espript.ibcp.fr) (Robert *et al*., 2014). (**B**) SDS-PAGE of wild-type (WT) RSV MTase-CTD and mutant proteins (that were successfully expressed) after Coomassie blue staining, purified by affinity chromatography. Molecular weights (in kilodaltons) are shown on the left. (**C**) The effect of point mutations (see panel A) in the RSV MTase-CTD on the MTase activity was analyzed by filter-binding assay. Samples were purified by affinity chromatography. GpppG-RSV_9_ was used as substrate and the RSV MTase-CTD was used at 25 nM. Data are the mean ± SEM of three independent measurements.
(TIF)

S2 Fig MTase activity reaction optimization. (**A**) Methylation activity of the RSV MTase-CTD protein measured by filter-binding assay using GpppG-RSV_9_ RNA as template at different pH (from 6.0 to 9.0). Plotted values were obtained after 3 h incubation at 30°C. The RSV MTase-CTD was used at the concentration of 25 nM. Data are the mean ± SEM of three independent measurements. (**B**) Methylation activity of the RSV MTase-CTD protein (25 nM) measured as in (A) in the standard reaction buffer (50 mM Tris-HCl; pH 8.0) or with different additives. Plotted values were obtained after 4 h incubation at 30°C. Data are the mean ± SEM of three independent measurements.
(TIF)

S3 Fig Inhibition of the MTase activity by cap analogues. Increasing concentrations of (**A**) unmethylated (Gppp A, Gppp G) and (**B**) N7-methylated (^m^Gppp A, ^m^GDP) cap analogues (previously dissolved in water) were incubated with 25 nM RSV MTase-CTD in a reaction mixture (40 mM Tris-HCl, pH 7.5, 2 μM SAM and 0.1 μM ^3^H-SAM) in the presence of 0.7 μM of GpppG-RSV_9_ synthetic RNA. Reactions were incubated at 30°C for 3 h. Values were normalized and fitted with GraphPad Prism version 5.0 using the following equation: Y = 100/(1+((X/IC_50_)^Hillslope)) (n = 3; mean value ± SEM).
(TIF)

S1 Table Liste of synthetic RNAs. Unmethylated caps are denoted by ‘Gppp’, N7-methylated caps by ‘mGppp’, 2’-*O*-methylated residues by ‘Xm’. Full-length sequences can be found in the second column, manufacturing information in the third one.
(TIF)

S2 Table Liste of primers. Primers were used for site-directed mutagenesis of RSV MTase-CTD protein by PCR amplification using the Pfu DNA polymerase kit (Promega).
(TIF)
