# Supplementary figures and images for: The methyltransferase domain of the Respiratory Syncytial Virus L protein catalyzes cap N7 and 2’-O-methylation
Source: PLoS Pathog. 2021 May 6;17(5):e1009562. doi: 10.1371/journal.ppat.1009562 (PMC8130918; doi:10.1371/journal.ppat.1009562)

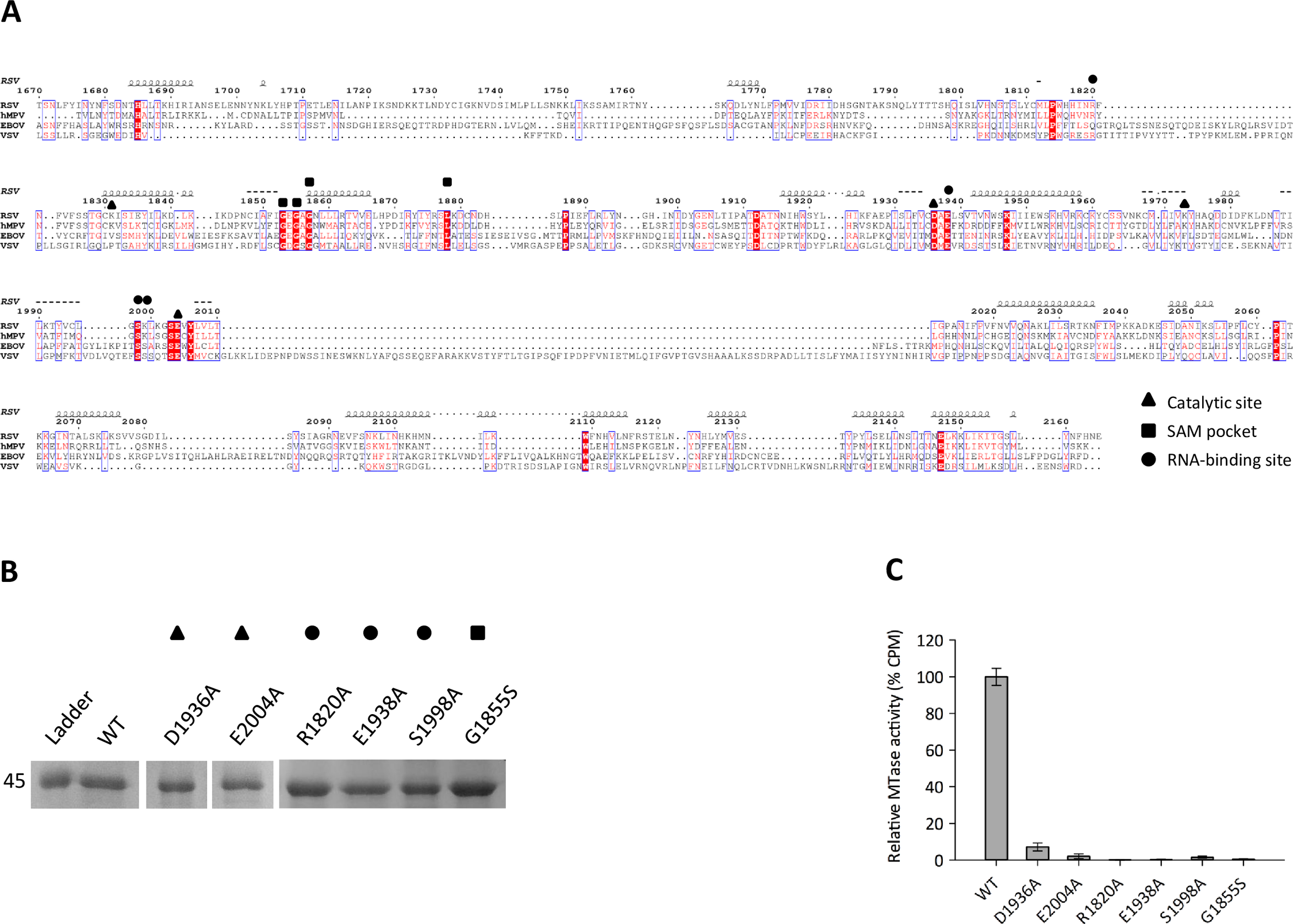

Supplement: S1 Fig — (A) Amino acid sequence alignment of the C-terminal portion of the indicated L proteins showing the amino acid residues of the catalytic tetrad (K-D-K-E) and the amino acid residues thought to participate in SAM and RNA-binding that were mutated. The amino acid sequences of the L protein from RSV strain A2 (P28887), human metapneumovirus (Q91L20), Zaire ebolavirus strain Mayinga-76 (Q05318) and vesicular stomatitis virus Indiana strain (P03523.2) were aligned using Seaview (Gouy et al., 2010) and ESPript (http://espript.ibcp.fr) (Robert et al., 2014). (B) SDS-PAGE of wild-type (WT) RSV MTase-CTD and mutant proteins (that were successfully expressed) after Coomassie blue staining, purified by affinity chromatography. Molecular weights (in kilodaltons) are shown on the left. (C) The effect of point mutations (see panel A) in the RSV MTase-CTD on the MTase activity was analyzed by filter-binding assay. Samples were purified by affinity chromatography. GpppG-RSV9 was used as substrate and the RSV MTase-CTD was used at 25 nM. Data are the mean ± SEM of three independent measurements. (TIF) [file ppat.1009562.s001.tif]

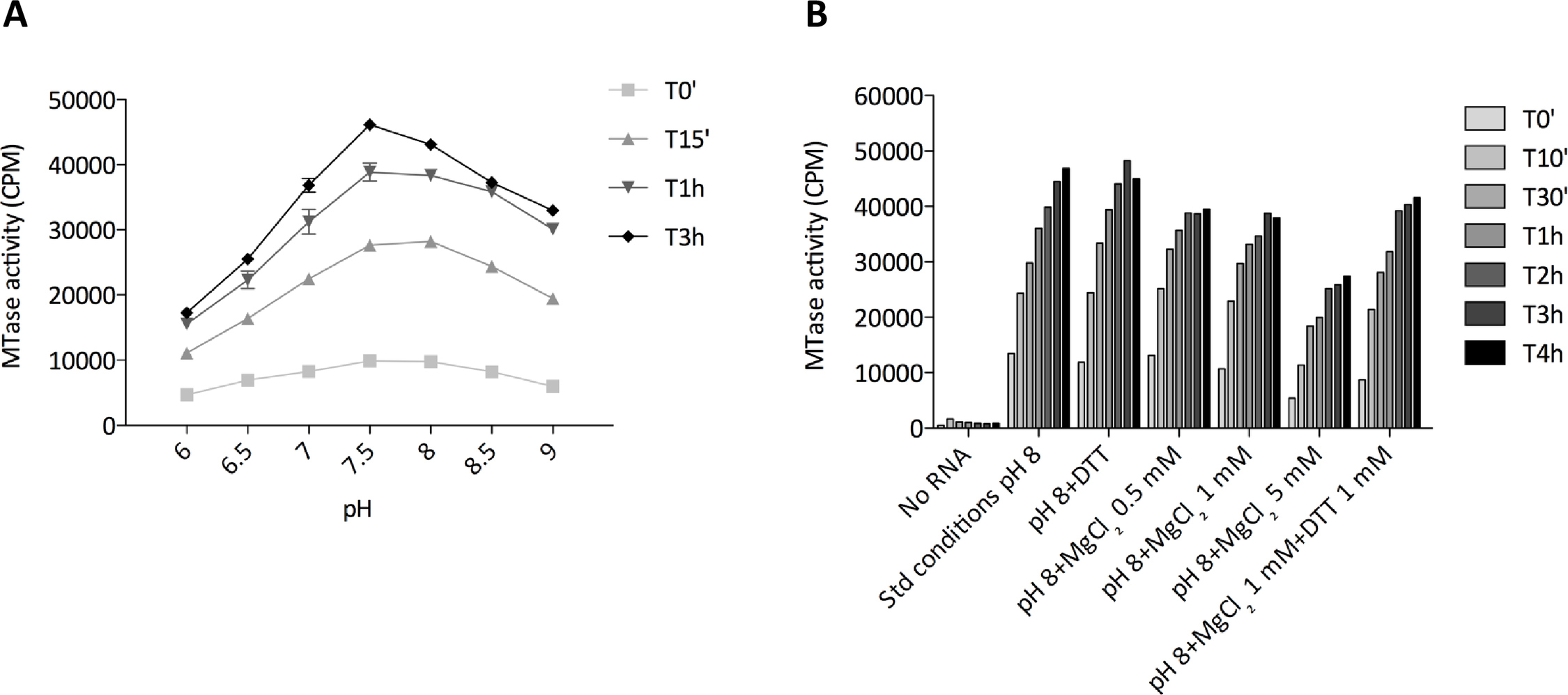

Supplement: S2 Fig — (A) Methylation activity of the RSV MTase-CTD protein measured by filter-binding assay using GpppG-RSV9 RNA as template at different pH (from 6.0 to 9.0). Plotted values were obtained after 3 h incubation at 30°C. The RSV MTase-CTD was used at the concentration of 25 nM. Data are the mean ± SEM of three independent measurements. (B) Methylation activity of the RSV MTase-CTD protein (25 nM) measured as in (A) in the standard reaction buffer (50 mM Tris-HCl; pH 8.0) or with different additives. Plotted values were obtained after 4 h incubation at 30°C. Data are the mean ± SEM of three independent measurements. (TIF) [file ppat.1009562.s002.tif]

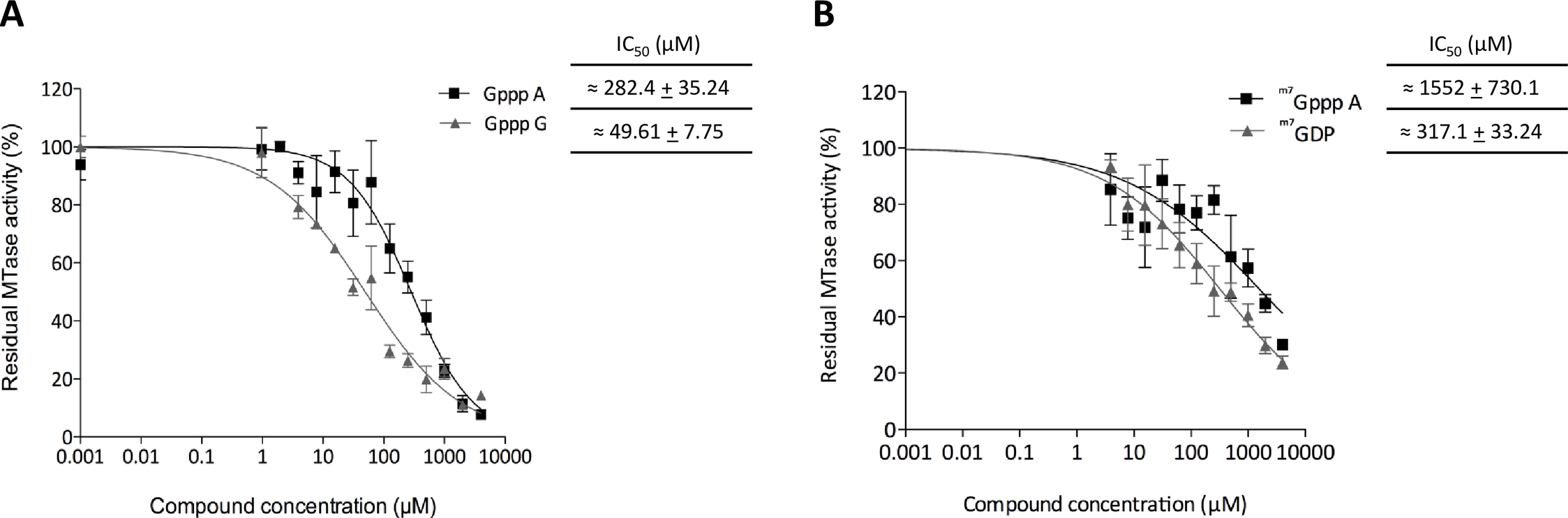

Supplement: S3 Fig — Increasing concentrations of (A) unmethylated (Gppp A, Gppp G) and (B) N7-methylated (mGppp A, mGDP) cap analogues (previously dissolved in water) were incubated with 25 nM RSV MTase-CTD in a reaction mixture (40 mM Tris-HCl, pH 7.5, 2 μM SAM and 0.1 μM 3H-SAM) in the presence of 0.7 μM of GpppG-RSV9 synthetic RNA. Reactions were incubated at 30°C for 3 h. Values were normalized and fitted with GraphPad Prism version 5.0 using the following equation: Y = 100/(1+((X/IC50)^Hillslope)) (n = 3; mean value ± SEM). (TIF) [file ppat.1009562.s003.tif]

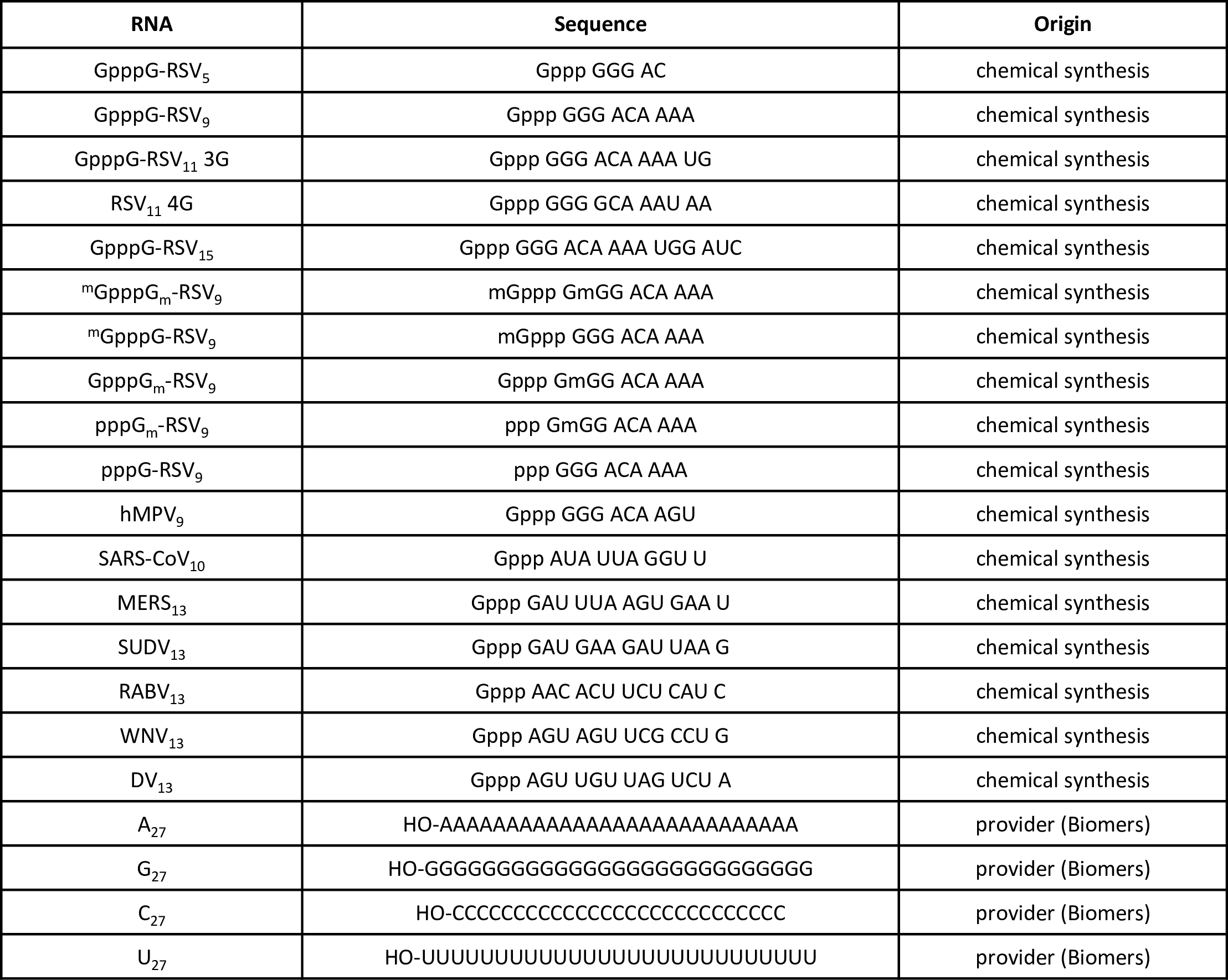

Supplement: S1 Table — Unmethylated caps are denoted by ‘Gppp’, N7-methylated caps by ‘mGppp’, 2’-O-methylated residues by ‘Xm’. Full-length sequences can be found in the second column, manufacturing information in the third one. (TIF) [file ppat.1009562.s004.tif]

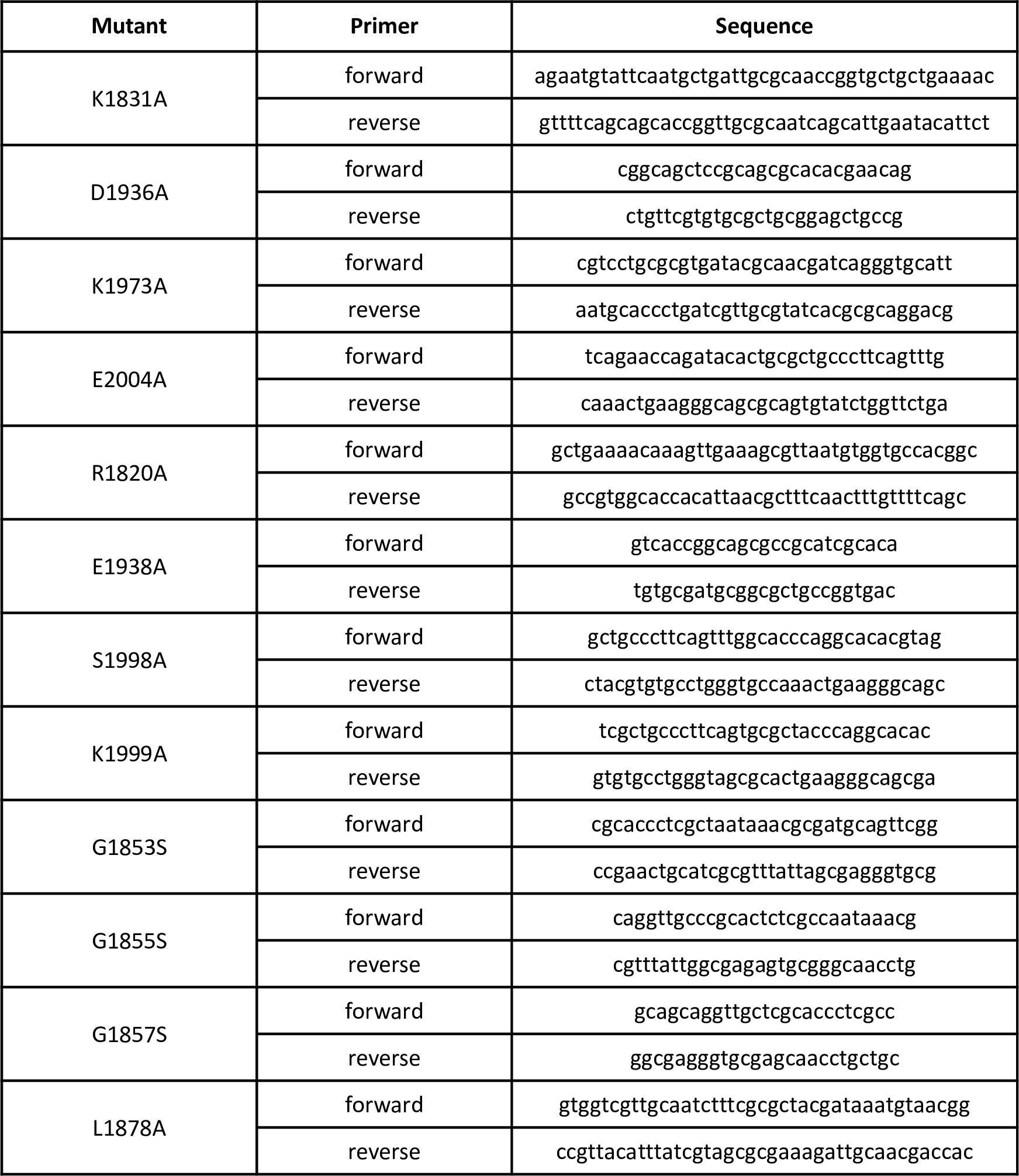

Supplement: S2 Table — Primers were used for site-directed mutagenesis of RSV MTase-CTD protein by PCR amplification using the Pfu DNA polymerase kit (Promega). (TIF) [file ppat.1009562.s005.tif]
